# Unraveling the connection between gut microbiota and Alzheimer’s disease: a two-sample Mendelian randomization analysis

**DOI:** 10.3389/fnagi.2023.1273104

**Published:** 2023-10-16

**Authors:** Huiqiong Zeng, Kaixia Zhou, Yu Zhuang, Aidong Li, Baiwei Luo, Ye Zhang

**Affiliations:** ^1^Department of Rheumatology, Shenzhen Futian Hospital for Rheumatic Diseases, Shenzhen, Guangdong, China; ^2^Department of Clinical Laboratory, Shanghai Cancer Center, Fudan University, Shanghai, China; ^3^Department of Rheumatology and Immunology, Huizhou Central People’s Hospital, Huizhou, Guangdong, China; ^4^Department of Rehabilitation, The Second People’s Hospital of Futian District, Shenzhen, Guangdong, China; ^5^Department of Rheumatology and Immunology, Yuebei People’s Hospital Affiliated to Shantou University Medical College, Shaoguan, Guangdong, China; ^6^Department of Traditional Chinese Medicine, Women and Children Health Institute Futian Shenzhen, Shenzhen, China

**Keywords:** Mendelian randomization, gut microbiota, Alzheimer’s disease, causal relationship, genetic analysis

## Abstract

**Purpose:**

Studies have shown a close relationship between gut microbiota (GM) and Alzheimer’s disease (AD). However, the causal relationship between them remains unclear.

**Methods:**

We conducted a genome-wide association study (GWAS) using publicly available summary statistics data for GM and AD. We extracted independent genetic loci significantly associated with GM relative abundances as instrumental variables based on predefined thresholds (*p* < 1*e−5). The inverse variance-weighted (IVW) method was primarily used for causal relationship assessment. Additional analyses, including MR-Egger, weighted median, simple mode, and weighted mode, were performed as supplementary analyses.

**Results:**

IVW analysis revealed significant correlations between certain microbial taxa and the risk of AD. Higher abundances of *Actinobacteria* at the class level, phylum. *Actinobacteria*, class. *Deltaproteobacteria*, order. *Desulfovibrionales*, genus. *Oscillospira*, and genus. *Ruminococcaceae* UCG004 (*p* < 0.048) was found to be positively associated with an elevated risk of AD. However, within the genus-level taxa, *Ruminococcus1* (*p* = 0.030) demonstrated a protective effect on lowering the risk of AD. In addition, to ensure the robustness of the findings, we employed Cochrane’s *Q* test and leave-one-out analysis for quality assessment, while the stability and reliability of the results were validated through MR-Egger intercept test, MR-PRESSO global test, and sensitivity analysis.

**Conclusion:**

This study provided a comprehensive analysis of the causal relationship between 211 GM taxa and AD. It discerned distinct GM taxa linked to the susceptibility of AD, thereby providing novel perspectives on the genetic mechanisms governing AD via the GM. Additionally, these discoveries held promise as valuable biomarkers, enabling the identification of potential therapeutic targets and guiding forthcoming AD investigations.

## 1. Background

The gut microbiota (GM) is a diverse microbial community in the human body, primarily composed of bacteria, viruses, protozoa, archaea, and fungi. It weighs 1.5 kg, with bacteria being the dominant group. About 70% of the microbiota resides in the gastrointestinal tract, forming a symbiotic relationship with the host (Zhao, 2013). Factors such as delivery mode, diet, and disease influence its composition (Conlon and Bird, 2014). GM plays a crucial role in nutrient metabolism and produces neuroactive compounds (Morais et al., 2021). Imbalances in the GM are associated with diseases like inflammatory bowel disease and diabetes (Lavelle and Sokol, 2020; Iatcu et al., 2021). The gut-brain axis facilitates bidirectional communication between the microbiota and the central nervous system (Morais et al., 2021). The GM is essential for overall health and is implicated in neurological disorders like Alzheimer’s disease (AD) (Jiang et al., 2017).

AD is a common neurodegenerative disorder characterized by cognitive impairment, predominantly affecting the elderly population. The onset of the disease is insidious and slow, with a progressive worsening of symptoms (Lane et al., 2018). The latest data from the Centers for Disease Control and Prevention (CDC) published in Alzheimer’s Association (2022) indicated an increasing mortality rate with affecting nearly 6.5 million people for AD in the United States (Alzheimer’s Association, 2022). This places a significant psychological and economic burden on families of individuals with AD and exerts a substantial economic pressure on society as a whole. The typical histopathological changes observed in AD involve abnormal accumulation of extracellular β-amyloid protein (Aβ) forming senile plaques and intracellular hyperphosphorylated tau protein leading to the formation of neurofibrillary tangles (NFTs) within neurons (d'Errico and Meyer-Luehmann, 2020). The exact pathogenic mechanisms of AD remain unclear; however, research has demonstrated notable differences in the GM of AD patients compared to healthy individuals, indicating significant alterations in its composition.

Research indicated that the GM and its metabolites induce the release of downstream inflammatory factors by activating the nuclear factor-κB (NF-κB) signaling pathway and nucleotide-binding domain and leucine-rich repeat containing protein 3 (NLRP3) inflammasome formation, leading to the generation of neuroinflammation, ultimately damaging neurons and affecting the onset of AD (Sun et al., 2022). In the context of the cholinergic hypothesis, GM alterations, specifically a decrease in *lactobacilli* in the gut, could result in a corresponding reduction in acetylcholine (Ach) levels. The decrease in Ach leaded to dysregulation of cholinergic neurotransmitter function in the cortex, which forms the basis of cognitive impairment in AD patients (Hira et al., 2019). AD had been reported to be associated with infections caused by herpes simplex virus, spirochetes, *Chlamydia pneumoniae*, and fungi (Piekut et al., 2022). Moreover, several gut bacterial strains, including *Escherichia coli*, *Bacillus subtilis*, *Salmonella enterica*, *Enterococcus faecalis*, *Mycobacterium tuberculosis*, and *Staphylococcus aureus*, are capable of producing abundant amyloid-like proteins. The abnormal accumulation and folding of these proteins contribute to the pathological changes involved in AD, particularly the formation of amyloid-beta (Aβ) plaques (Megur et al., 2020). Preliminary observations in the amyloid precursor protein/presenilin 1 (APP/PS1) transgenic mouse model of AD had revealed an increase in *Rikenellaceae* and a decrease in *Allobaculum* and *Akkermansia* species (Stephenson et al., 2018). Establishing research on the relationship between GM and AD is necessary because different study designs can lead to different conclusions, and the human gastrointestinal tract is influenced by various factors, including diet and rest. Therefore, there may be a causation between them.

The objective of this study is to investigate the causal relationship between GM and AD by utilizing two-sample Mendelian randomization (TSMR) analysis. Mendelian randomization (MR) is a statistical method used in epidemiological inference to elucidate causal relationships between exposures and outcomes (Bowden and Holmes, 2019). It employs genetic variations associated with the exposure as instrumental variables (IVs) (Woolf et al., 2022). MR is an epidemiological investigative tool that assesses causal relationships between exposure factors and outcomes by leveraging genetic variations. Unlike observational studies, genetic variations form at birth and remain stable, rendering the associations derived from MR more robust. MR enhances etiological research efficiency, gaining prominence in post-genetic era epidemiology. It’s easier than costly clinical trials, utilizing genome-wide data to study millions of genetic influences on traits, potentially uncovering complex disease causes. MR analysis has been widely applied in various disease studies, thanks to the significant advancements in large-scale genome-wide association studies (GWAS) (Xu et al., 2022; Gibson et al., 2023; Jiang et al., 2023). However, the mechanisms underlying the application of MR analysis to unravel the causal relationship between GM and AD have not been thoroughly investigated. Previous observational studies have laid the groundwork by establishing general associations and shedding some light on the intricate relationship between GM and AD. Nevertheless, a more profound understanding of this connection and the underlying regulatory mechanisms within specific genetic contexts demands more comprehensive investigation. Notably, this study marks a pioneering effort in the field, presenting the inaugural MR analysis focused on discerning the causal relationship between GM and AD. This innovative approach was propelled by the ongoing quest to unravel the multifaceted mechanisms driving AD, with the ultimate goal of unraveling the intricate interplay between GM and the disease. By establishing a causal connection, our study introduces new theoretical underpinnings that could pave the way for future intervention strategies targeting AD.

## 2. Materials and methods

### 2.1. Study design

The exposure factor was defined as the presence of 211 GM taxa, while the outcome was represented by AD in this study. Causal relationship between GM and AD was assessed through a two-sample MR analysis. Initially, GWAS data associated with GM and AD were obtained. The MR analysis was conducted based on three classical assumptions: (1) the selected IVs were strongly associated with the exposure factor, (2) the IVs were independent of any confounding factors, and (3) the IVs influenced the outcome solely through the pathway of GM exposure. Furthermore, adherence to the STROBE-MR guidelines was ensured throughout the study (Skrivankova et al., 2021).

### 2.2. Sources of exposure data

**Figure 1:**
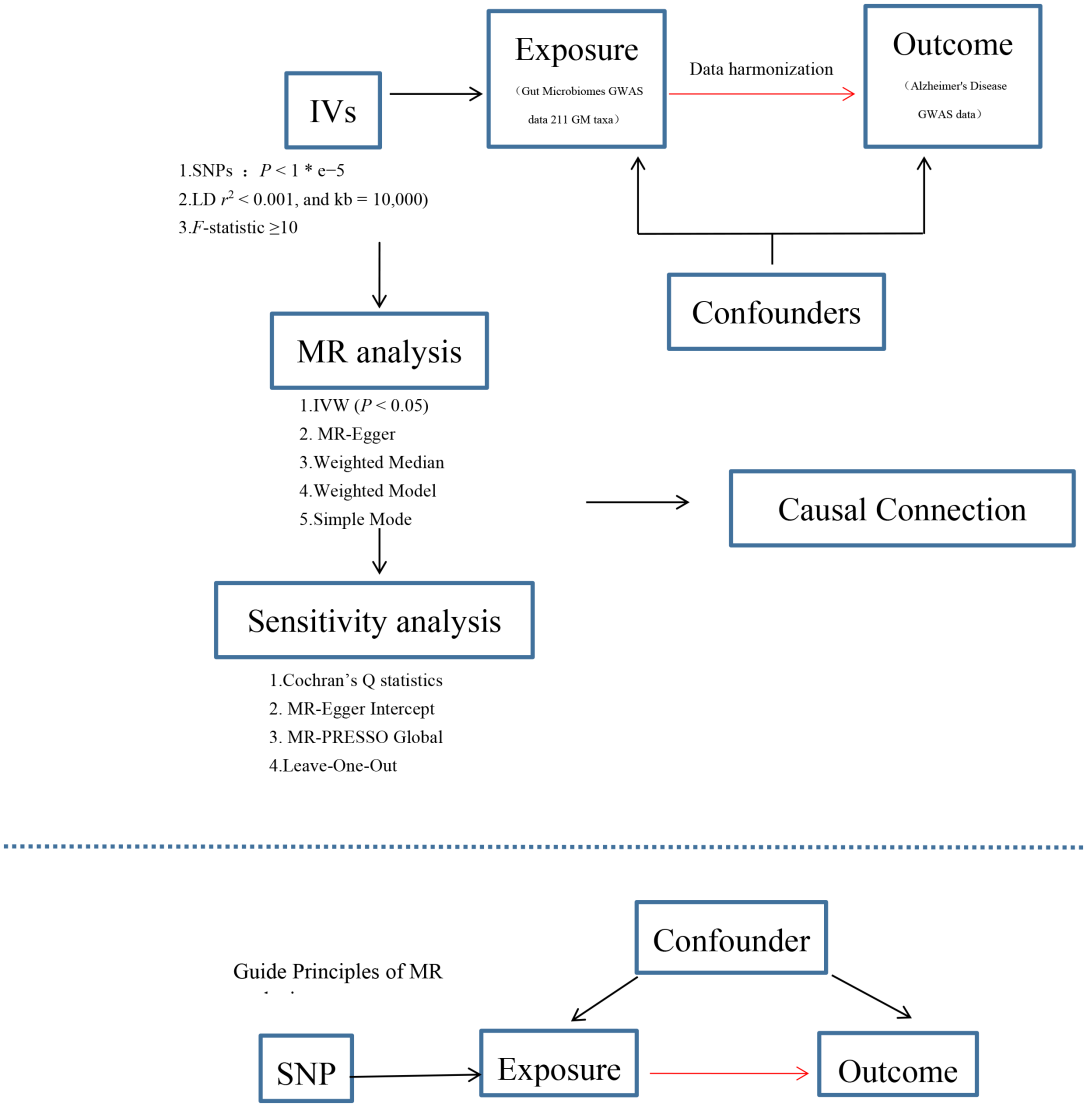
Flow chat of this work.

Genetic data of the GM was obtained from the latest large-scale GWAS meta-analysis data (MiBioGen consortium),^1^ including 18,340 individuals from 24 cohorts. This GWAS study examined 211 transgenic taxa (from phylum to genus) and 122,110 associated single nucleotide polymorphism (SNPs) (Kurilshikov et al., 2021). The GM composition was identified using three different variable regions of the 16S rRNA gene (V4, V3-V4, and V1-V2) and genetic variations influencing the relative abundance of microbial taxa were identified through the localization of microbiome quantitative trait loci (mbQTL). Given that all statistical analyses relied on existing publicly available summary data, no additional ethical approval was required. Figure 1 illustrated the entire flow chat of this study.

### 2.3. Outcome data collection

We obtained summary statistics data for late-onset AD from the eighth edition of the FinnGen consortium’s publicly available GWAS.^2^ This prospective cohort study included a total of 342,499 participants as of March 2023. Using the IEU database with “Alzheimer’s disease” as the keyword for searching, we identified 15 relevant GWAS studies and compiled relevant information. We selected one GWAS study with a more recent publication year and a larger number of SNPs, which included 63,926 AD patients and 10,528,610 controls (Kunkle et al., 2019). The diagnosis of AD was based on the International Classification of Diseases code (ICD-10: F00). For more information with FinnGen on the above, please refer to https://r8risteys.finngen.fi/.

### 2.4. Instrumental variables identification

Based on the previous investigations, the objective of this work was to evaluate the causal association between GM and IVs (*p* < 1*e−5). The methodology employed in this study was consistent with previous investigations (VanderWeele et al., 2014). The specific screening steps conducted were as follows: Relevant SNPs were extracted using the TwoSampleMR package in *R* software (version 4.2.0); SNPs were subjected for clustering analysis to mitigate the impact of linkage disequilibrium (LD). The LD differentiation ability of MR surpassed other methods. SNPs with LD status were identified using two parameters: LD clustering algorithm (*r*^2^) and kb values. SNPs with an *r*^2^ threshold of <0.001 and a window size of 10,000 kb were selected; Echo SNPs were excluded from the analysis; The remaining SNPs were derived from the initial SNP pool after undergoing value of *p* filtering, chain imbalance removal, and echo sequence elimination; The PhenoScanner database^3^ was utilized to retrieve significant results from GWAS data regarding genomic correlations (*p* < 1*e−5) (Yavorska and Burgess, 2017). Furthermore, confounding factors were accounted for excluding; To avoid bias from weak instruments, the IVs strength for each bacterial taxon was estimated using the *F*-statistic (Burgess and Thompson, 2011). With the increase of *F*-statistic value, the deviation will decrease. The formula of *F*-statistic is *R*^2^ (*N*–2)/(1–*R*^2^), see detail in Supplementary Table 1.

### 2.5. Statistical and sensitivity analysis

In this study, we utilized the TSMR method to assess the potential causal relationship between GM and AD. The primary analytical methods used were the inverse variance weighted (IVW), MR-Egger, Weighted median (WM), Simple Mode, and Weighted Mode methods. Robust analysis was conducted to avoid the influence of outliers. Cochran’s Q and *I*^2^ values were computed to assess heterogeneity among the estimated SNP effects. IVs with *p*-values less than 0.05 were considered heterogeneous (Kennedy et al., 2020).

The pleiotropic analysis is as follows: the IVW method obtains the overall effect estimate by weighted averaging of all IVs, assuming no horizontal pleiotropy bias. The WM method estimates the causal relationship by taking the median of the genetic IVs, which is more robust and mitigates the influence of outliers. The MR-Egger intercept test is used to assess the horizontal pleiotropy and SNP-level pleiotropy. If the intercept term in the MR-Egger intercept test is statistically significant, it indicates substantial horizontal pleiotropy in the MR analysis (Bowden et al., 2015).

The Mendelian Randomization Pleiotropy Residual Sum and Outlier (MR-PRESSO) method was used to used to validate the results of the IVW model and correct for the influence of outliers (Verbanck et al., 2018). We performed leave-one-out analysis by systematically removing each SNP and repeating the IVW analysis to assess the consistency of the causal effect driven by individual SNPs (Cai et al., 2022). These methods require adherence to the underlying assumptions and employ various analytical techniques to correct for various biases and confounders, including the removal of genetic variants that exhibit pleiotropy based on biological or statistical evidence. Statistical analysis was performed using *R* Studio and relevant packages, and the results were labeled as *p* < 0.001. Funnel plots and forest plots were constructed for visualizing the presence of horizontal pleiotropy in the MR analysis (Cai et al., 2022).

## 3. Result

### 3.1. Screening selection of IVs and MR analysis outcomes

According to the aforementioned steps, we selected a total of 2,506 SNPs as IVs for 211 GM taxa. By applying screening criteria based on *p* < 1*e−5 and LD analysis thresholds, a total of 7 GM taxa were identified. This study is not influenced by weak instrument bias (*F*-statistic value ranged from = 15.91 ~ 114.65). After matching the data from GM and AD, a final set of 88 SNPs were included in the TSMR study. Considering the potential confounding factors, we used Phenoscanner to search for SNPs associated with the aforementioned confounders, but found no significant associations.

### 3.2. MR analysis between GM and AD

**Figure 2:**
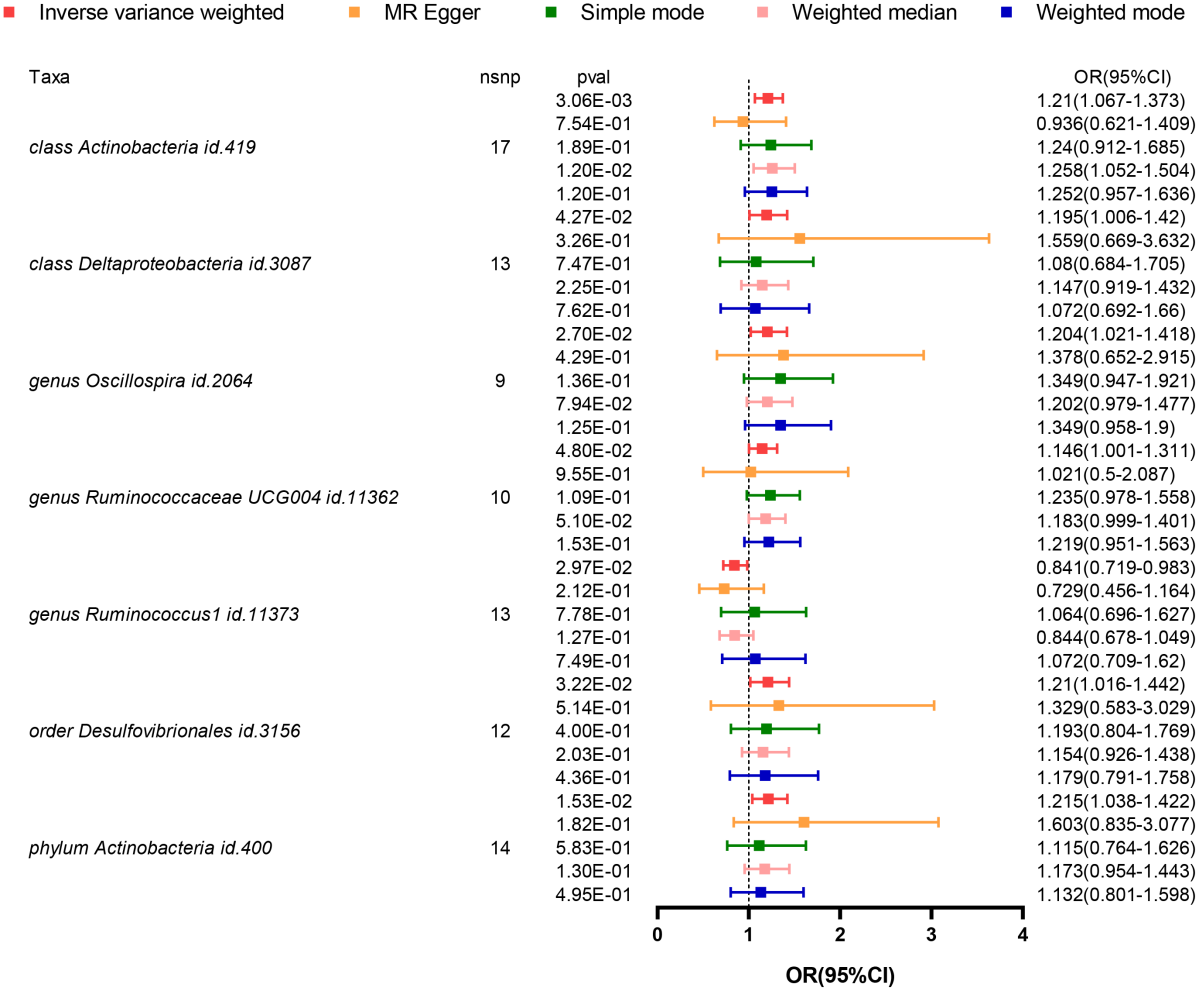
Forest plot visualizing the results of MR methods to analyze the causal relationship between GM and the risk of AD. GM, Gut Microbiota; AD, Alzheimer’s Disease; IVs, Instrumental variables; MR, Mendelian randomization; GWAS, Genome-wide association study; SNP, Single nucleotide polymorphism; LD, Linkage disequilibrium; IVW, Inverse variance weighted.

Based on the results from five different methods of MR, as shown in Figure 2; Table 1, at least one method observed 7 causal relationships between the GM features (1 phylum, 2 class, 1 order, and 3 genus) and AD traits. The results of the MR analysis using the IVW method with a random-effects model were as follows: an increased relative abundance of the *Actinobacteria* class (OR = 1.210, 95%CI: 1.067–1.373, *p* = 0.003), *Actinobacter* phylum (OR = 1.215, 95%CI: 1.038–1.422, *p* = 0.015), *Deltaproteobacteria* class (OR = 1.195, 95%CI: 1.006–1.420, *p* = 0.043), *Desulfovibrionales* order (OR = 1.210, 95%CI: 1.016–1.442, *p* = 0.032), *Oscillospira* genus (OR = 1.204, 95%CI: 1.021–1.418, *p* = 0.027), and *Ruminococcaceae* UCG004 genus (OR = 1.146, 95%CI: 1.001–1.311, *p* = 0.048) was found to be correlated with an elevated risk of AD. However, within the genus-level taxa, *Ruminococcus1* genus (OR = 0.841, 95%CI: 0.719–0.983, *p* = 0.030) demonstrated a protective effect on lowing the risk of AD.

**Table 1:** Genetic connection for GM vs. AD by five methods (*p <* 1*e−5).

Exposure group	GM	NSNP	MR methods	SE	*p* value	MR effect		
						OR	95% LCI	95% UCI
Class	*Actinobacteria*	17	Inverse variance weighted	0.064	0.003	1.210	1.067	1.373
			MR Egger	0.209	0.754	0.936	0.621	1.409
			Simple mode	0.157	0.189	1.240	0.912	1.685
			Weighted median	0.091	0.012	1.258	1.052	1.504
			Weighted mode	0.137	0.120	1.252	0.957	1.636
	*Deltaproteobacteria*	13	Inverse variance weighted	0.088	0.043	1.195	1.006	1.420
			MR Egger	0.432	0.326	1.560	0.670	3.632
			Simple mode	0.233	0.747	1.080	0.684	1.705
			Weighted median	0.113	0.225	1.147	0.919	1.432
			Weighted mode	0.223	0.762	1.071	0.692	1.660
Genus	*Oscillospira*	9	Inverse variance weighted	0.084	0.027	1.204	1.021	1.418
			MR Egger	0.382	0.429	1.378	0.652	2.915
			Simple mode	0.180	0.136	1.349	0.947	1.921
			Weighted median	0.105	0.079	1.202	0.979	1.477
			Weighted mode	0.175	0.125	1.349	0.958	1.900
	*Ruminococcaceae UCG004*	10	Inverse variance weighted	0.069	0.048	1.146	1.001	1.311
			MR Egger	0.365	0.956	1.021	0.500	2.087
			Simple mode	0.119	0.110	1.235	0.979	1.558
			Weighted median	0.086	0.051	1.183	0.999	1.401
			Weighted mode	0.127	0.153	1.219	0.951	1.563
	*Ruminococcus1*	13	Inverse variance weighted	0.080	0.030	0.841	0.719	0.983
			MR Egger	0.240	0.212	0.729	0.456	1.164
			Simple mode	0.217	0.778	1.064	0.696	1.627
			Weighted median	0.111	0.127	0.844	0.678	1.049
			Weighted mode	0.211	0.749	1.072	0.709	1.620
Order	*Desulfovibrionales*	12	Inverse variance weighted	0.090	0.032	1.210	1.016	1.442
			MR Egger	0.420	0.514	1.329	0.583	3.029
			Simple mode	0.201	0.400	1.193	0.804	1.769
			Weighted median	0.112	0.203	1.154	0.9257	1.438
			Weighted mode	0.204	0.436	1.179	0.791	1.759
Phylum	*Actinobacteria*	14	Inverse variance weighted	0.080	0.015	1.215	1.038	1.422
			MR Egger	0.333	0.182	1.603	0.835	3.077
			Simple mode	0.193	0.583	1.115	0.764	1.626
			Weighted median	0.105	0.130	1.173	0.954	1.443
			Weighted mode	0.176	0.500	1.132	0.801	1.598

GM, Gut Microbiota; AD, Alzheimer’s disease; MR, Mendelian randomization; SNP, Single nucleotide polymorphism.

### 3.3. Sensitivity analysis

In these seven causal associations, all *I*^2^ values in the Cochran’s Q heterogeneity test are less than 50%, and all *p*-values are greater than 0.05, indicating no evidence of heterogeneity (Table 2). Furthermore, according to the results of the MR-Egger regression intercept analysis, there is no significant directional pleiotropy in each taxa (*p* > 0.05). The MR-PRESSO global analysis demonstrates no evidence of horizontal pleiotropy (global test *p* > 0.05). The leave-one-out plot does not show any significant influence on the overall results when removing any individual SNP, confirming the credibility of the MR results (Supplementary Figure 1). The funnel plot demonstrates a relatively symmetrical distribution of the various instrumental variables with minimal bias (Supplementary Figure 2). As shown in the scatter plot, the calculated directions are consistent for all taxa, except for the *Actinobacteria* class and *Ruminococcus1* genus (Supplementary Figure 3).

**Table 2:** Sensitivity analysis for GM vs. AD by MR analysis.

Exposure		Horizontal pleiotropy		Heterogeneity	Steiger	MR-PRESSO global test
		MR egger-interpreter	MR egger-interpreter *p* value	Cochran’s *Q* *p* value	*p* value	*p* value
Class	*Actinobacteria*	0.019	0.214	0.724	1.37E-64	0.756
	*Deltaproteobacteria*	−0.018	0.542	0.183	1.92E-39	0.215
Genus	*Oscillospira*	−0.013	0.726	0.289	9.02E-28	0.332
	*Ruminococcaceae UCG004*	0.010	0.757	0.855	6.61E-34	0.888
	*Ruminococcus1*	0.011	0.539	0.501	1.11E-45	0.513
Order	*Desulfovibrionales*	−0.010	0.824	0.223	3.73E-37	0.25
Phylum	*0.*	−0.018	0.408	0.289	2.50E-53	0.307

GM, Gut Microbiota; AD, Alzheimer’s disease; MR, Mendelian randomization.

## 4. Discussion

In this study, we utilized Mendelian Randomization (MR) design to explore the causal relationship between gut microbiota (GM) and the risk of Alzheimer’s disease (AD). Genetic predictions indicated a positive correlation between *Actinobacteria*, *Bacteroidetes*, *Desulfobacterales*, *Oscillospira*, and *Ruminococcaceae* UCG004 and the risk of AD. Furthermore, we identified a protective causal effect of *Ruminococcus*1 on the pathogenesis of AD. These findings were consistently demonstrated through a series of sensitivity and quality control analyses.

In contemporary research, it has been discovered that the human gastrointestinal tract harbors over 4 trillion microorganisms, with a total gene count approximately 150 times greater than the number of genes in the human genome. Among these microorganisms, 99% are bacteria collectively known as the GM (Adak and Khan, 2019). There is mounting evidence suggesting a close correlation between the ecological imbalance of the GM and the intestinal mucosal immune system. Particularly in the context of AD research, it has been observed that AD is often associated with dysbiosis or alterations in the GM, making the concept of the gut-brain axis a prominent focus in this field.

The GM is involved in the pathogenesis of AD through various mechanisms. Simultaneously, the pathological conditions of AD may lead to changes in the GM (Angelucci et al., 2019). Therapeutic interventions such as probiotic supplementation and fecal microbiota transplantation (FMT) can improve AD by modulating the GM (Kim et al., 2020; Imbimbo and Watling, 2021). Probiotics, serving as psychobiotics, hold potential in regulating gastrointestinal and neural homeostasis. Probiotics and prebiotics, as dietary supplements, maintain health through enhancement of immunity, intestinal mucosal adhesion, and metabolite transport. Research indicates that probiotic interventions alter the fecal microbiota composition in individuals with AD, thereby sustaining gut-brain axis equilibrium (Abraham et al., 2019; Den et al., 2020). Studies reveal that probiotics significantly alleviate depressive emotions and improve mood related to various neurological disorders, including AD (Cheng et al., 2019). Probiotics enhance cognitive function in AD patients by reducing levels of inflammatory and oxidative biomarkers. A clinical trial has demonstrated that the novel anti-AD drug, GV-971, can restore GM and alleviate brain neuroinflammation (Bonfili et al., 2021). Furthermore, another study suggests that substantial utilization of probiotics can enhance cognitive abilities in AD mice (Xiao et al., 2021). Current research advancements underscore the potential of probiotics in modulating gut-brain axis disorders, offering promising new therapeutic avenues for individuals with AD.

Fecal Microbiota Transplantation (FMT) is a therapeutic approach wherein donor fecal material is transferred to a recipient to modulate the composition of the intestinal microbiota, aimed at treating diseases. Although FMT has demonstrated success in various gastrointestinal disorders, notably in cases of *Clostridium difficile* infection (Wang et al., 2019). A recent research has uncovered potential therapeutic prospects of FMT in the broadly used AD model: APP/PS double transgenic (Tg) mouse model. This approach exhibits the ability to mitigate cerebral Aβ deposition, regulate Tau protein phosphorylation, reduce levels of Aβ40 and Aβ42, enhance cognitive function, and promote synaptic plasticity (Sun et al., 2019). Another animal experiment has also substantiated the capacity of FMT to ameliorate cognitive decline and amyloid-like protein accumulation in AD mice (Cheng et al., 2023). Hang Z et al. observed that FMT treatment can reverse the GM remodeling in AD mice, also rectifying the metabolic aberrations of inorganic and organic salts in the intestinal microbiota of AD mice (Hang et al., 2022). Despite predominantly being investigated in animal models, FMT holds promise as a potential avenue for treating neurodegenerative disorders, particularly AD, through mechanisms encompassing restoration of Short-Chain Fatty Acids (SCFA) and disruption of Aβ oligomers in the future.

Encouragingly, *Clostridium butyricum* (CB), a butyrate-producing probiotic from the GM, has already undergone a clinical trial for AD treatment (Den et al., 2020).

In both case–control studies and observational researches, the determination of the exposure time and outcomes can be challenging, making them susceptible to confounding factors (Abraham et al., 2019). Few studies have conducted a comprehensive and genetic investigation at the species level between GM and AD. Therefore, investigating the causal relationship between the two not only deepens our understanding of the pathogenesis of AD but also facilitates the exploration of microbiological therapeutic interventions for AD in clinical practice. Consequently, it is imperative to elucidate the causal relationship between GM and different types of AD.

The combination of 16S rRNA gene sequencing and metabolomics provide a new perspective for understanding the relationship between GM and AD. Imbalance of the intestinal microbiota may be a key factor in the occurrence of AD. *Actinobacteria, Proteobacteria, Firmicutes*, and *Bacteroidetes* are the major bacterial phyla found in the human gastrointestinal tract (Morais et al., 2021). A cohort study of elderly AD patients in China revealed that, compared to the healthy control (HC) group, AD patients had reduced fecal microbial diversity and increased abundance of *Bacteroidetes* in the early stages of the disease. Furthermore, when compared to individuals with pre-onset amnestic mild cognitive impairment (aMCI), AD patients exhibited decreased levels of *Ruminococcus* at the phylum level in their fecal microbiota (Cheng et al., 2019), findings that are consistent with the conclusions of our study.

Researchers have found a significant association between the abundance of certain bacterial phyla at the genus level with AD. Subjects with higher abundance of *Actinobacteria* had a 1.16 times higher likelihood of developing AD compared to the HC group. Additionally, *Bacteroidetes* showed decreased abundance, and abnormal levels of *Ruminococcus* at the strain and species level were unique to AD patients (Bonfili et al., 2021).

Fragilis Bacteroides (BF) lipopolysaccharide can penetrate the blood–brain barrier (BBB) and enter the cytoplasm of neural cells, coupling with and significantly upregulating pro-inflammatory microRNA-146a (miRNA-146a) and microRNA-155 (miRNA-155). Each miRNA’s direct promoter contains multiple NF-kB DNA binding and activation sites, leading to neuro-inflammation (Xiao et al., 2021). When compared to the control group, AD subjects showed increased abundance of *Proteobacteria, Firmicutes, Actinobacteria*, and *Bacteroidetes* in brain tissue. Researchers observed variations in β diversity between hippocampal and cerebellar samples, suggesting the presence of brain microbiota (Wang et al., 2019). Xi et al. (2021) measured the fecal microbial and metabolic profiles of 21 AD individuals. Non-targeted GM classification was analyzed based on next-generation sequencing (NGS) of 16S ribosomal RNA genes, and fecal metabolites were quantified using ultra-high-performance liquid chromatography-mass spectrometry (UPLC-MS). The study revealed that AD participants exhibited increased abundance of *Ruminococcaceae* UCG-007 compared to non-AD participants (Sun et al., 2019). Conversely, in a study conducted in the United States, AD patients showed reduced abundance of *Firmicutes* and *Actinobacteria*, and increased abundance of *Bacteroidetes* and *Proteobacteria* (Cheng et al., 2023). The possible reasons for the inconsistency in the results of GM research include individual differences, technical variations, sample sources, dietary factors, age and lifecycle, disease status, and differences in reporting and interpretation. To reduce inconsistency, it is essential to employ rigorous research design, sample selection, and control for potential confounding factors.

The study discovered a possible association GM dysbiosis and AD risk, which may involve the following mechanisms: ① Gram-negative bacteria, including *Bacteroidetes*, in the GM produce endotoxins that activate macrophages, triggering an inflammatory response (Stilling et al., 2016; Hang et al., 2022). ② Imbalance or dysregulation of the GM can impact the levels of short-chain fatty acids (SCFAs) and worsen cerebral Aβ burden in APP/PS1 transgenic mice (Yadav and Lewis, 2021; Hang et al., 2022). ③ Bacterial product lipopolysaccharide (LPS) has recently been shown to be associated with the peripheral region of neuronal cell nuclei in sporadic AD, affecting the expression of amyloid precursor protein (APP) or its processing enzymes. High abundance of *Bacteroidetes* may be associated with the production of metabolites that promote APP cleavage, leading to increased Aβ generation, affecting Aβ aggregation and clearance in the brain, and further exacerbating AD pathology (Liu et al., 2019). These studies support the notion that GM may promote the development of AD by crossing the biophysical barriers.

The present study has significant strengths. Firstly, it represents the most comprehensive genetic research conducted to date investigating the causal relationship between the GM and AD, as it encompasses analyses of 211 bacterial taxa. Secondly, the rigorous TSMR analysis technique has been employed to address inherent biases and confounders observed in previous studies. The use of multiple methods and detailed analyses has enhanced the reliability and validity of the findings.

The study has made significant progress in exploring the potential causal relationship between the GM and AD. However, there are several limitations to consider. Firstly, the TSMR analysis only utilized GWAS data from European populations. Further investigations in diverse populations are warranted. Secondly, the inclusion of limited data on GM abundance hinders a comprehensive analysis of the causal association. Additional GWAS data on GM are necessary for a thorough exploration of the GM-AD link. Thirdly, while TSMR is an effective causal analysis method, validation through future animal experiments is essential to confirm the potential causal link between the AD and GM. Lastly, it is crucial to acknowledge that the relationship between the two is not solely a unidirectional causal relationship. A multi-dimensional approach is required to investigate the etiology and pathogenesis of AD comprehensively.

## 5. Conclusion

This study highlights the importance of GM composition in relation to AD risk. Further research is warranted to elucidate the underlying mechanisms and explore the potential of modulating the GM as a preventive or therapeutic strategy for AD.

Our study confirms the causal effect of GM on AD through TSMR. Specific strains like *Actinobacteria* at the family level, phylum. *Actinobacteria*, class. *Deltaproteobacteria*, order. *Desulfovibrionales*, genus. *Oscillospira*, and genus. *Ruminococcaceae* UCG004 are associated with AD and could be potential biomarkers. GMs such as within the genus-level taxa, *Ruminococcus1* may hold promise for AD prevention and treatment. This research provides genetic insights for managing AD.

## Data availability statement

The original contributions presented in the study are included in the article/supplementary material, further inquiries can be directed to the corresponding author.

## Ethics statement

Written informed consent was not obtained from the individual(s) for the publication of any potentially identifiable images or data included in this article because Given that all statistical analyses relied on existing publicly available summary data, no additional ethical approval was required. Figure 1 illustrated the entire flow chat of this study.

## Author contributions

HZ: Writing – review & editing, Conceptualization, Data curation, Formal analysis, Funding acquisition, Investigation, Methodology, Resources, Writing – original draft. KZ: Data curation, Formal analysis, Investigation, Methodology, Project administration, Resources, Supervision, Writing – original draft. YZhu: Data curation, Formal analysis, Investigation, Methodology, Project administration, Resources, Writing – original draft. AL: Conceptualization, Data curation, Investigation, Methodology, Project administration, Resources, Supervision, Writing – original draft. BL: Data curation, Formal analysis, Investigation, Methodology, Resources, Supervision, Writing – original draft. YZha: Data curation, Investigation, Methodology, Resources, Writing – review & editing.

## Footnotes

^1^www.mibiogen.org, accessed on 2 May 2023.

^2^https://www.finngen.fi/en, id: ieu-b-2, accessed on 2 May 2023.

^3^http://www.phenoscanner.medschl.cam.ac.uk/phenoscanner, accessed on May 2, 2023.
