# Supplementary material for: Unraveling the connection between gut microbiota and Alzheimer’s disease: a two-sample Mendelian randomization analysis
Source: Front Aging Neurosci. 2023 Oct 16;15:1273104. doi: 10.3389/fnagi.2023.1273104 (PMC10613649; doi:10.3389/fnagi.2023.1273104)
Supplement: Supplementary file 3 [file Data_Sheet_3.docx]

**Figure S1.** Leave-one-out analysis of each GM taxa on the risk of AD. The error line represents a 95% confidence interval. (A) class *Actinobacteria*. (B) class *Deltaproteobacteria*. (C) genus *Oscillospira*. (D) genus *Ruminococcaceae*. (E) genus *Ruminococcus 1*. (F) order *Desulfovibrionales*. (G) phylum *Actinobacteria*.

**Figure S2.** Funnel plots are utilized in sensitivity analysis to examine the causal relationship between GM and the risk of AD. When there is no heterogeneity, the funnel plot exhibits a symmetric shape, indicating that there is no systematic relationship between the study effects and their precision. These plots use different lines to represent distinct statistical methods. The X-axis represents the estimated values of causal effects, while the Y-axis represents the reciprocal of the standard errors of correlation. Black dots represent each SNP, the deep blue line represents the estimate using the IVW method, and the light blue line represents the estimate using the MR Egger method.

**Figure S3.** Scatter plots of sensitivity analysis for the causal relationship between GM and the risk of AD. The Y-axis displays the associations between these identical SNPs and the outcome (AD). The vertical lines represent the 95% confidence intervals for the effect size on AD, while the 95% confidence intervals for the effect size on the taxa level are displayed as horizontal lines. The lines in each plots illustrate different statistical methods: Inverse variance-weighted method (red line), MR Egger method (green line), Simple mode (blue line), weighted median (yellow line), and weighted mode (purple line).

Abbreviate: GM Gut Microbiota; AD Alzheimer's Disease; IVW Inverse Variance Weighted;
