# Supplementary material for: Unraveling the connection between gut microbiota and Alzheimer’s disease: a two-sample Mendelian randomization analysis
Source: Front Aging Neurosci. 2023 Oct 16;15:1273104. doi: 10.3389/fnagi.2023.1273104 (PMC10613649; doi:10.3389/fnagi.2023.1273104)
Supplement: Supplementary file 4 [file Image_1.PDF]

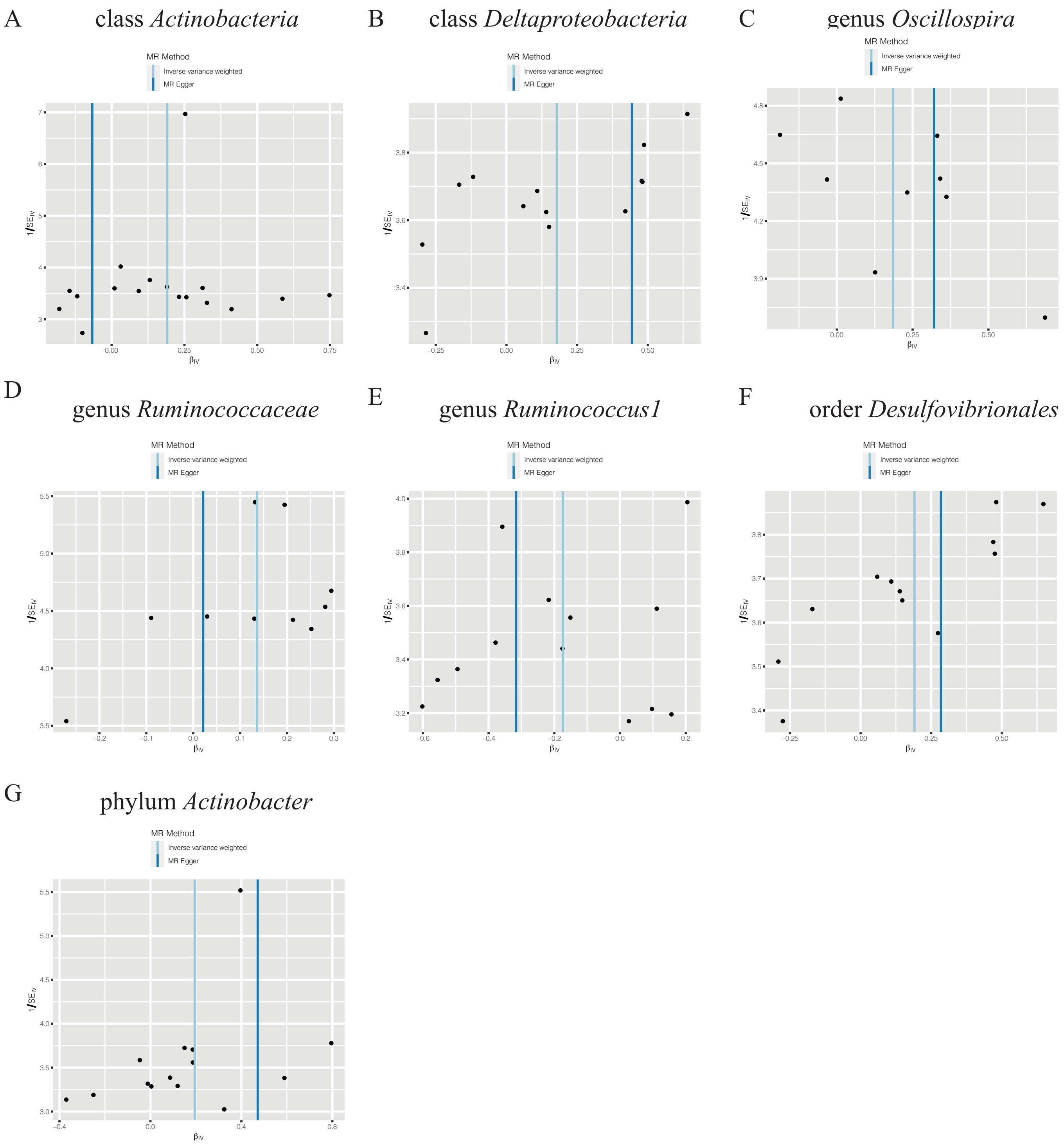
A
class Actinobacteria
B
class Deltaproteobacteria
C
genus Oscillospira
D
genus Ruminococcaceae
E
genus Ruminococcus1
F
order Desulfovibrionales
G
phylum Actinobacter
MR Method
Inverse variance weighted
MR Egger
1/SE_IV
β_IV
