# Supplementary figures and images for: Unraveling the connection between gut microbiota and Alzheimer’s disease: a two-sample Mendelian randomization analysis
Source: Front Aging Neurosci. 2023 Oct 16;15:1273104. doi: 10.3389/fnagi.2023.1273104 (PMC10613649; doi:10.3389/fnagi.2023.1273104)

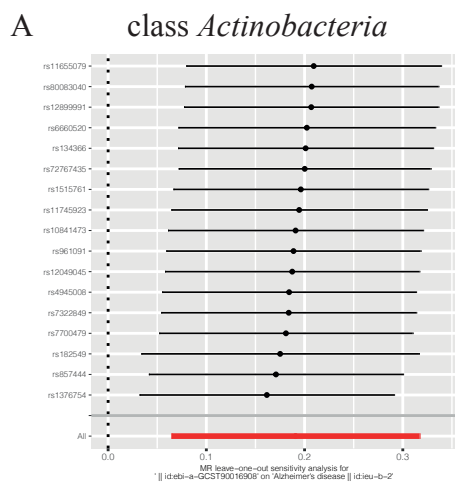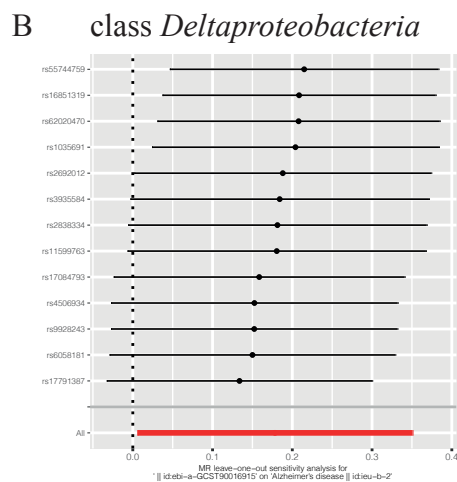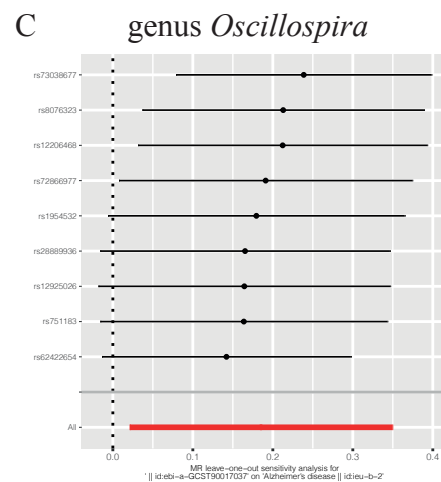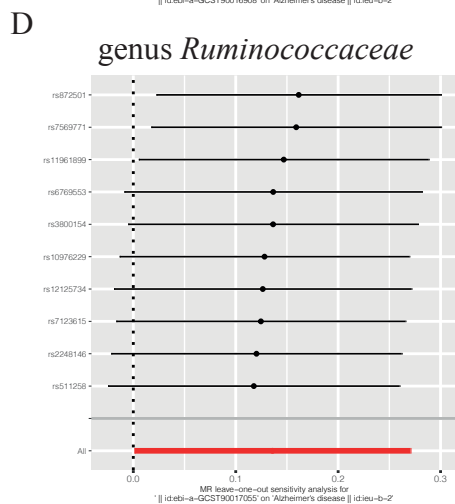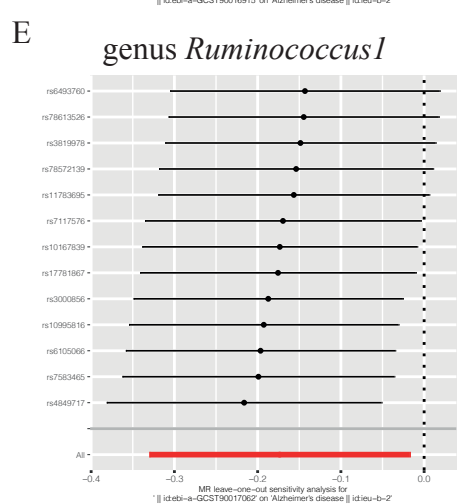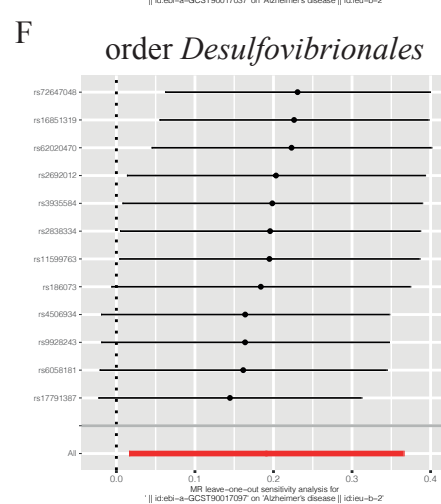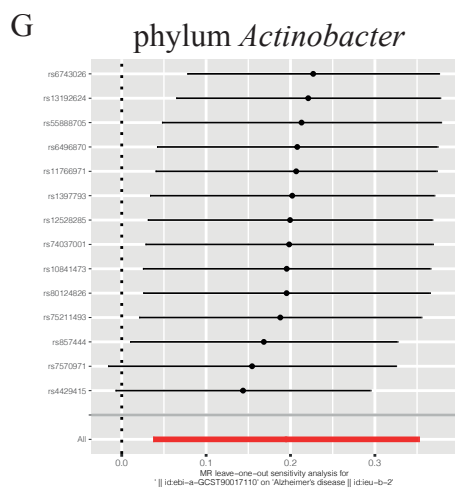

Supplement: Supplementary file 5 [file Image_2.PDF]
