# Supplementary material for: Unraveling the connection between gut microbiota and Alzheimer’s disease: a two-sample Mendelian randomization analysis
Source: Front Aging Neurosci. 2023 Oct 16;15:1273104. doi: 10.3389/fnagi.2023.1273104 (PMC10613649; doi:10.3389/fnagi.2023.1273104)
Supplement: Supplementary file 6 [file Image_3.PDF]

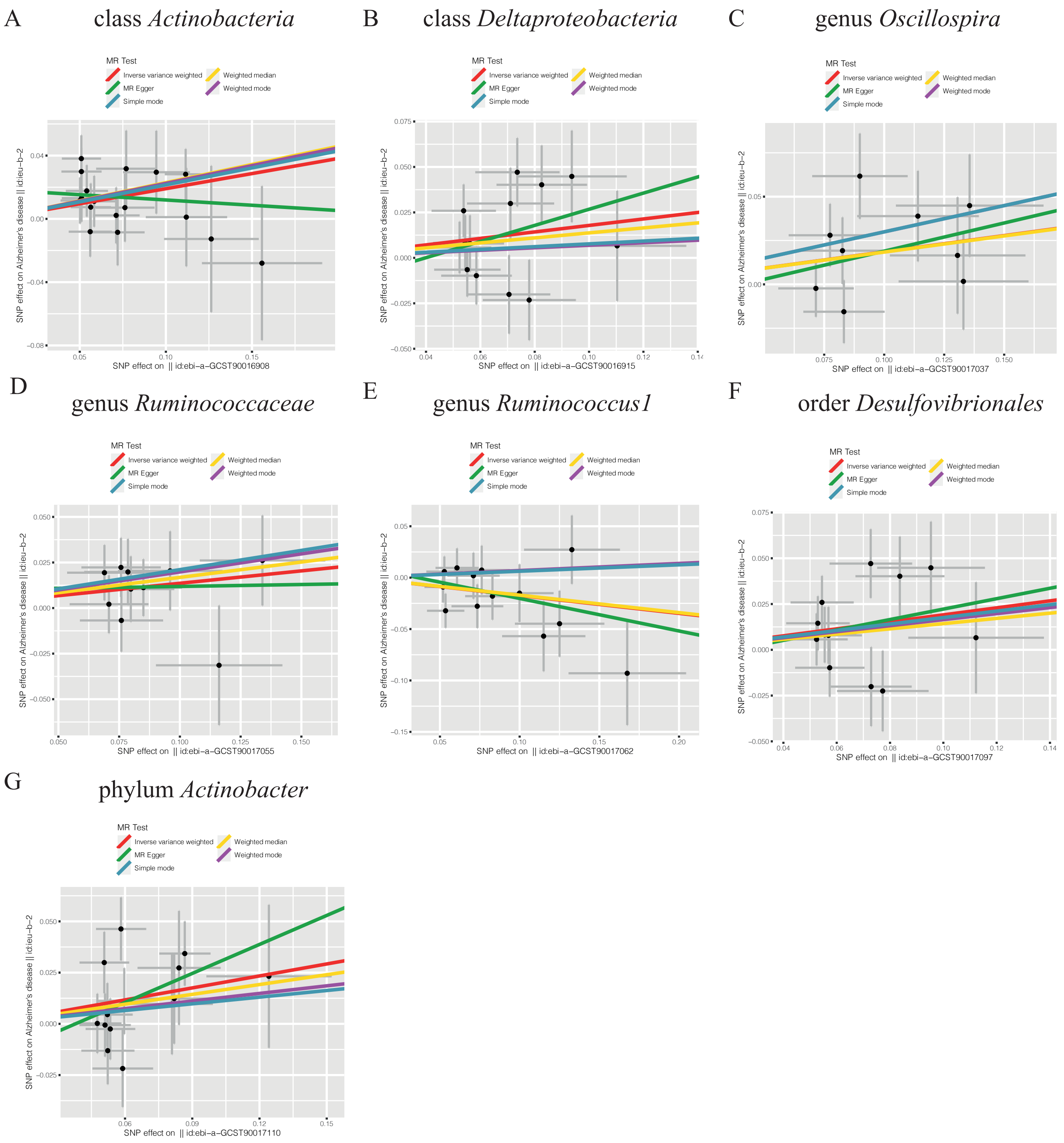
A
class *Actinobacteria*
MR Test
Inverse variance weighted
MR Egger
Simple mode
Weighted median
Weighted mode
SNP effect on Alzheimer's disease || id:ieu-b-2
SNP effect on || id:ebi-a-GCST90016908
B
class *Deltaproteobacteria*
MR Test
Inverse variance weighted
MR Egger
Simple mode
Weighted median
Weighted mode
SNP effect on Alzheimer's disease || id:ieu-b-2
SNP effect on || id:ebi-a-GCST90016915
C
genus *Oscillospira*
MR Test
Inverse variance weighted
MR Egger
Simple mode
Weighted median
Weighted mode
SNP effect on Alzheimer's disease || id:ieu-b-2
SNP effect on || id:ebi-a-GCST90017037
D
genus *Ruminococcaceae*
MR Test
Inverse variance weighted
MR Egger
Simple mode
Weighted median
Weighted mode
SNP effect on Alzheimer's disease || id:ieu-b-2
SNP effect on || id:ebi-a-GCST90017055
E
genus *Ruminococcus1*
MR Test
Inverse variance weighted
MR Egger
Simple mode
Weighted median
Weighted mode
SNP effect on Alzheimer's disease || id:ieu-b-2
SNP effect on || id:ebi-a-GCST90017062
F
order *Desulfovibrionales*
MR Test
Inverse variance weighted
MR Egger
Simple mode
Weighted median
Weighted mode
SNP effect on Alzheimer's disease || id:ieu-b-2
SNP effect on || id:ebi-a-GCST90017097
G
phylum *Actinobacter*
MR Test
Inverse variance weighted
MR Egger
Simple mode
Weighted median
Weighted mode
SNP effect on Alzheimer's disease || id:ieu-b-2
SNP effect on || id:ebi-a-GCST90017110
